# Short-peptide-based enteral nutrition affects rats MDP translocation and protects against gut-lung injury via the PepT1-NOD2-beclin-1 pathway in vivo

**DOI:** 10.1007/s11033-024-09759-0

**Published:** 2024-08-07

**Authors:** Xiu-feng Pang, Xiao-yong Dai, Lu-jia Zhao, You-wen Ye, Xiao-ying Yang, Huan-huan Wang, Meng Jiang, Yu-qin Zhu, Bin Shi

**Affiliations:** 1 Department of Emergency Intensive Care Unit, Yangpu Hospital, School of Medicine, Tongji University, No. 450, Tengyue Road, Shanghai, 200090 China; 2 Department of Geriatrics, Xinhua Hospital Affiliated to Shanghai Jiaotong University School of Medicine, Shanghai, 200092 China

**Keywords:** Muramyl dipeptide (MDP), Peptides, Inflammation, Mitochondria, Autophagy, Apoptosis

## Abstract

**Background:**

Peptide transporter 1 (PepT1) transports bacterial oligopeptide products and induces inflammation of the bowel. Nutritional peptides compete for the binding of intestinal bacterial products to PepT1. We investigated the mechanism of short-peptide-based enteral nutrition (SPEN) on the damage to the gut caused by the bacterial oligopeptide product muramyl dipeptide (MDP), which is transported by PepT1. The gut-lung axis is a shared mucosal immune system, and immune responses and disorders can affect the gut-respiratory relationship.

**Methods and results:**

Sprague-Dawley rats were gavaged with solutions containing MDP, MDP + SPEN, MDP + intact-protein-based enteral nutrition (IPEN), glucose as a control, or glucose with GSK669 (a NOD2 antagonist). Inflammation, mitochondrial damage, autophagy, and apoptosis were explored to determine the role of the PepT1-nucleotide-binding oligomerization domain-containing protein 2 (NOD2)-beclin-1 signaling pathway in the small intestinal mucosa. MDP and proinflammatory factors of lung tissue were explored to determine that MDP can migrate to lung tissue and cause inflammation. Induction of proinflammatory cell accumulation and intestinal damage in MDP gavage rats was associated with increased NOD2 and Beclin-1 mRNA expression. IL-6 and TNF-α expression and apoptosis were increased, and mitochondrial damage was severe, as indicated by increased mtDNA in the MDP group compared with controls. MDP levels and expression of proinflammatory factors in lung tissue increased in the MDP group compared with the control group. SPEN, but not IPEN, eliminated these impacts.

**Conclusions:**

Gavage of MDP to rats resulted in damage to the gut-lung axis. SPEN reverses the adverse effects of MDP. The PepT1-NOD2-beclin-1 pathway plays a role in small intestinal inflammation, mitochondrial damage, autophagy, and apoptosis.

## Introduction

Translocation of intestinal bacteria and their products may lead to enterogastric and systemic infections, and become the origin of many mediators of the inflammatory process, leading to systemic inflammation and even organopathy [1, 2]. Peptide transporter 1 (PepT1), a brush border oligopeptide transporter of the intestinal epithelium, transports not only short peptides (e.g., dipeptides and tripeptides), which are the products of protein decomposition, but also short peptide products of bacteria, such as MDP [3]. MDP is the minimum component that remains biologically potent of Peptidoglycan (PGN), which is a thin cell wall component of Gram-negative bacteria and a thick cell wall component of Gram-positive bacteria. MDP comprises MurNAc and two amino acids, D-Ala and D-isoGln (or D-Glu) [4]. In 1974, it was discovered that MDP was the minimal structure required for the efficacy of Freund’s Complete Adjuvant (FCA), and Ellouz F isolated natural N-acetylmuramyl-tripeptides from *M. smegmatis* and of *E. coli* were active [5]. White PJ and Gilvarg C prepare peptidoglycan from *Bacillus megaterium M46* [6]. A key site where bacterial products interact with the host and regulate epithelial regeneration is the intestinal crypt, lined by adult stem cells, PAN cells, and dividing epithelial cells. MDP causes inflammatory damage to the intestinal mucosa by stimulating the aggregation of inflammatory cells, which is delivered to intestinal epithelial cells via PepT1 [7].

The family of nucleotide-binding oligomerization domain (NOD) proteins, which includes NOD1 and NOD2, are important intracellular pattern recognition receptors in the epithelial cells of the intestine and immune cells [8], which play a major role in the intestinal cellular immune balance and autophagy [9, 10]. NOD2 perceives and combines with short peptide products of pathogenic microorganisms, namely pathogen-associated molecular patterns and damage-associated molecular patterns, after ischemic stress to initiate the immune response, which is the link to connect adaptive immunity, triggering a series of immune activations and inflammatory reactions that constitute the intestinal immune barrier [11, 12]. NOD2 has the characteristic of inhibiting inflammatory reactions. MDP is also a ligand of NOD2 [13]. The degree of inflammatory reactions in the abdominal cavity of infected rats with NOD2 gene knockout is much higher than that in normal rats. NOD2-mediated signaling elicits negative feedback inhibition against TLR4-mediated inflammatory responses. Rats with NOD2 gene mutations are more prone to the intestinal inflammatory injury and inflammatory bowel disease [9, 10, 14]. However, it is unclear whether MDP produces toxic effects on cells through other mechanisms. Our previous study showed that a short peptide (Gly-Gly) affects intestinal translocation of bacterial short peptide products to protect intestinal mucosa through the PepT1-NOD2 pathway. The PepTl-NOD2-Rip2-NF-кB signaling pathway might play a major role in bacterial product transport and activation of the intestinal inflammatory response [5].

NF-κB positively regulates beclin-1 expression [15]. Beclin-1 is an ortholog of the yeast Atg6 in mammals and plays a crucial role in autophagy, a programmed cell survival process that increases during cellular stress. It interacts with a number of co-factors (including Atg14L, PINK, VMP1, HMGB1, UVRAG, Bif-1, Rubicon, Ambra1, nPIST, SLAM, IP3R, and Survivin) to regulate the lipid kinase Vps-34 and promote the formation of the beclin-1-Vps34-Vps15 core complex to induce autophagy. Apoptosis-promoting BH3-only proteins, such as Bad, Bid, BNIP3, Noxa, and Puma, competitively bind to the Bcl-2/BxL B protein BH3 receptor groove with beclin-1 to increase apoptosis and autophagy [16]. The anti-apoptotic protein Bcl-2 has an autophagic inhibition function [17]. Beclin-1 may be at the center of autophagy, apoptosis, and inflammation in a complex network of cellular responses.

Researches have shown that intestinal flora is closely associated with gastrointestinal diseases, immune diseases, metabolic diseases, etc., they have an impact on the respiratory system [18, 19]. Starting with intestinal flora to solve respiratory diseases has become a new idea. The intestine-lung axis is a shared mucosal immune system, especially in respiratory immunity and anti-infection response, and the immune response and disorder can affect the relationship between the gut and the respiratory tract [20].

But it is unclear whether SPEN has the aforementioned effect of improving the intestinal inflammatory response and whether SPEN protects lung tissue through the gut-lung axis. Therefore, we hypothesize that SPEN may protect the intestinal mucosa from translocated MDP-induced inflammation through the NOD2-beclin-1 pathway, regulate intestinal epithelial autophagy, and protect lung tissue through the gut-lung axis.

## Materials and methods

### Animals and treatments

Male Sprague-Dawley rats (200–250 g) have been made available by the Tongji University Medical College Animal Experimentation Centre. The rats were randomly subdivided into five groups (*n* = 6):

(1) control, (2) MDP (15 mg/kg), (3) MDP (15 mg/kg) with IPEN (188.1 KJ/day), (4) MDP (15 mg/kg) with SPEN (188.1 KJ/day), (5) MDP (15 mg/kg), and injected GSK669 (NOD2 antagonist, 3.2 µM). Groups are blended using dextrose to ensure consistent calories in each group. Peptides were administered by gavage three times, with an interval of four hours. Rats were fed regular food and kept free in a 22 °C, 12-hour light/dark room for one week. The experiment was started after 24 h of fasting. Rats were gavaged with a 16 G FST18061-10 gavage needle and sacrificed four hours after the last gavage. Rats’ tail veins were used to extract peripheral blood into tubes containing EDTA prior to the animals being put to death. After centrifugation at 10,000 g for 3 min, serum samples were gathered and saved below 20 °C until analyzed. After putting the rats to death, lung lobes and jejunum section tissue were collected and saved as above for analysis. This study was approved by the Animal Ethics Committees of Yangpu Hospital and Tongji University (LL-2022-WSJ-005). It was conducted in accordance with the guidelines for the Care and Use of Laboratory Animals of the National Institutes of Health.

### Enzyme-linked immunosorbent assays (ELISAs)

Serums and supernatants obtained by homogenization and centrifugation from lung tissues were used for the assay. The proinflammatory factors IL-6, TNF-α, and MDP were quantified by means of ELISA kits (BioLegend, USA) in accordance with the producer’s manual, and determined optical density on a Multiskan Spectrum at 450 nm (Bio-Tek, China).

### Histological analysis

Jejunum mucosa sections were excised, fixed with paraformaldehyde, dehydrated, and paraffin embedded. The sections were stained with hematoxylin and eosin, and the morphology was examined by an optical microscope (CX41, Tokyo, Japan) at ×80 magnification. The stained jejunal segments were graded histologically based on Chiu’s intestinal mucosal injury grading by a pathologist specializing in pathology using a double-blind method. 1. Grade 0 - Mucosal villi are normal. Grade 1 - Subepithelial interstitial space develops, usually at the tip of the villi; typically escorted by capillary hemorrhage. Grade 2 - The subepithelial space is expanded, and the epithelial layer is moderately separated from the intrinsic layer. Grade 3 - The epithelium on either side of the villi is extensively elevated. Grade 4 - The villi are depressed, and the lamina propria and dilated capillaries are exposed. Lamina propria cells increased. Grade 5 - Digestion and breakdown of membranes propria; bleeding and ulcers [21].

### qPCR analysis

Total RNA was extracted from the mucosa membranes of jejunal segments using TRIzol (Invitrogen, USA) according to the producer’s manual. A QIAamp DNA Mini Kit (Qiagen, Germany) was used to extract total mtDNA from jejunal segments mucosal membranes according to the manufacturer’s protocol. The first Strand cDNA Synthesis Kit (Invitrogen, USA) was used to reverse transcribed Beclin-1, NOD2, and PepT1 mRNA into cDNA. The qRT-PCR was carried out using a Quantity Nova SYBR Green PCR Kit (Qiagen) and a 480II Real-Time PCR System (Roche). The following primers were used:

β-actin: forward, 5ʹ-tgccactcagaagactgtgg-3ʹ;

reverse, 5ʹ-ttcagctctgggatgacctt-3ʹ;

Beclin-1: forward, 5ʹ-cggctcctattccatcaaaa-3ʹ;

reverse, 5ʹ-ccacttgagattcgtcagca-3ʹ;

NOD2: forward, 5ʹ-gtgtttggggctgtcagaag-3ʹ;

reverse, 5ʹ-ggtgggatgaagggagtgaa-3ʹ;

PepT1: forward, 5ʹ-aaacgtgtcttctcccggat-3ʹ;

reverse, 5ʹ-gttctcccctttctctggct-3ʹ;

mtDNA: forward, 5ʹ-CGGCTCCTTCTCCCTACAAA-3ʹ;

reverse, 5ʹ-TTGGTCAGGCGGGGATTAAT-3ʹ.

The 2-ΔΔCt method was used to calculate target gene expression.

### Western blot assay

Protein buffer (Beyotime, China) was used to extract total proteins from the mucosal membranes of the jejunal segments. A BCA protein quantitative kit (Sigma, USA) was used to determine protein concentration. The proteins that are extracted were separated by 7.5% sodium dodecyl sulphate-polyacrylamide gel electrophoresis, transferred to polyvinylidene fluoride membranes (Millipore, USA), then blocked with 7.5% dry fat-free milk overnight, followed by incubation with primary antibodies against NOD2, beclin-1, PepT1, LC3-II, or GAPDH (1:1,500 dilution; Abcam, USA) for 1 h at room temperature (primary antibodies purchased from Shanghai Ribiology Biotech Co. with order no. QD2205220263). The membranes were incubated with secondary antibodies (1:5,000; Abcam) for 1 h at room temperature after washing in Tris-buffered saline with Tween-20. Protein bands were developed using an advanced chemiluminescence detection kit (Best Bio, China) according to the manufacturer’s instructions.

### TUNEL staining

Paraffin-embedded sections were rehydrated and stained with a TUNEL Kit (Roche, Japan) according to the manufacturer’s protocol. Following washing, the jejunal segments have been counterstained with DAPI and coated with an anti-fluorescent burst sealer. Under an inverted fluorescence microscope (Nikon, Japan), the sections were observed and imaged at 500 times magnification.

### Statistical analysis

All statistical analyses were performed with GraphPad Prism version 9.4.1 for Windows. Results are expressed as the mean ± SD. The ordinary one-way ANOVA test was used to compare significant differences among means (*P* < 0.05), followed by Tukey’s multiple comparison post hoc test. Statistical significance was accepted at *P* < 0.05.

## Results

### SPEN improves small intestinal damage caused by MDP

**Fig. 1:**
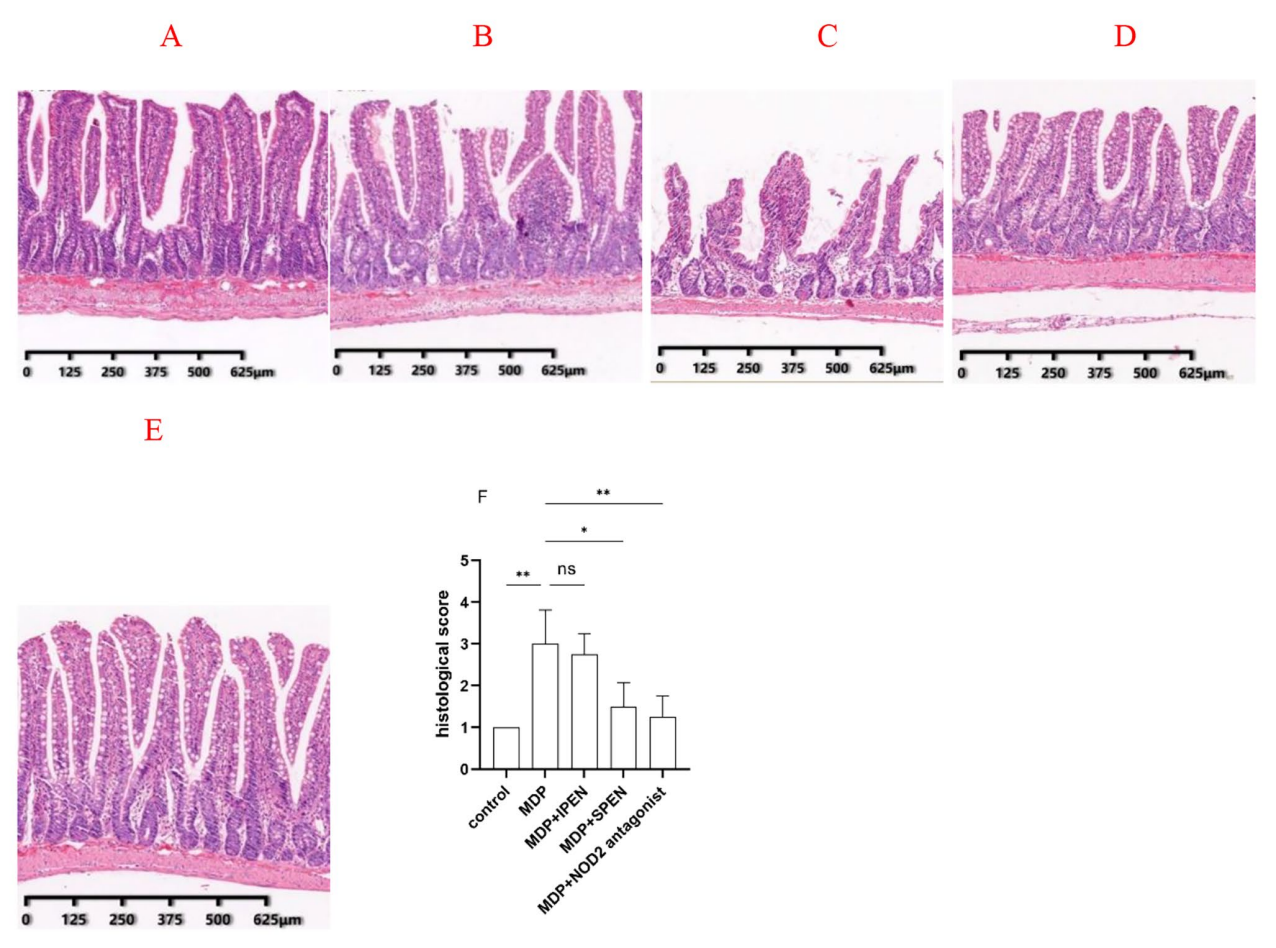
**A-E**: Histopathological changes in the jejunal tissue of rats (80×, hematoxylin and eosin stain). **A**: control group, **B**: MDP group, **C**: MDP with IPEN group; **D**: MDP with SPEN group; **E**: MDP with NOD2 antagonist group. **F**: The histology score was calculated as follows. Data were expressed as mean ± SD, *n* = 6; *P < 0.05; **P < 0.01; ***P < 0.001; ****P < 0.0001

As shown in Fig. 1A, the control group had well defined epithelial structures. In addition, compared to the control group, we observed more severe mucosal injury in the MDP group (Fig. 1B). Anyway, mucosal injury was improved in the MDP group compared to the control group. in the MDP with SPEN group (Fig. 1D), but not in the MDP with IPEN group (Fig. 1C). The jejunal mucosal tissue damage was also less severe in the MDP with NOD2 antagonist group than in the MDP group (Fig. 1E). The MDP with SPEN group had a significantly reduced histological score compared with the MDP group (Fig. 1F). Similarly, the histological score was significantly lower in the NOD2 antagonist group compared to the MDPs. The results showed that SPEN attenuates the microscopic damage in the gut following MDP-induced injury through the inhibition of NOD2.

### SPEN reduces the inflammatory response in plasma caused by MDP

**Fig. 2:**
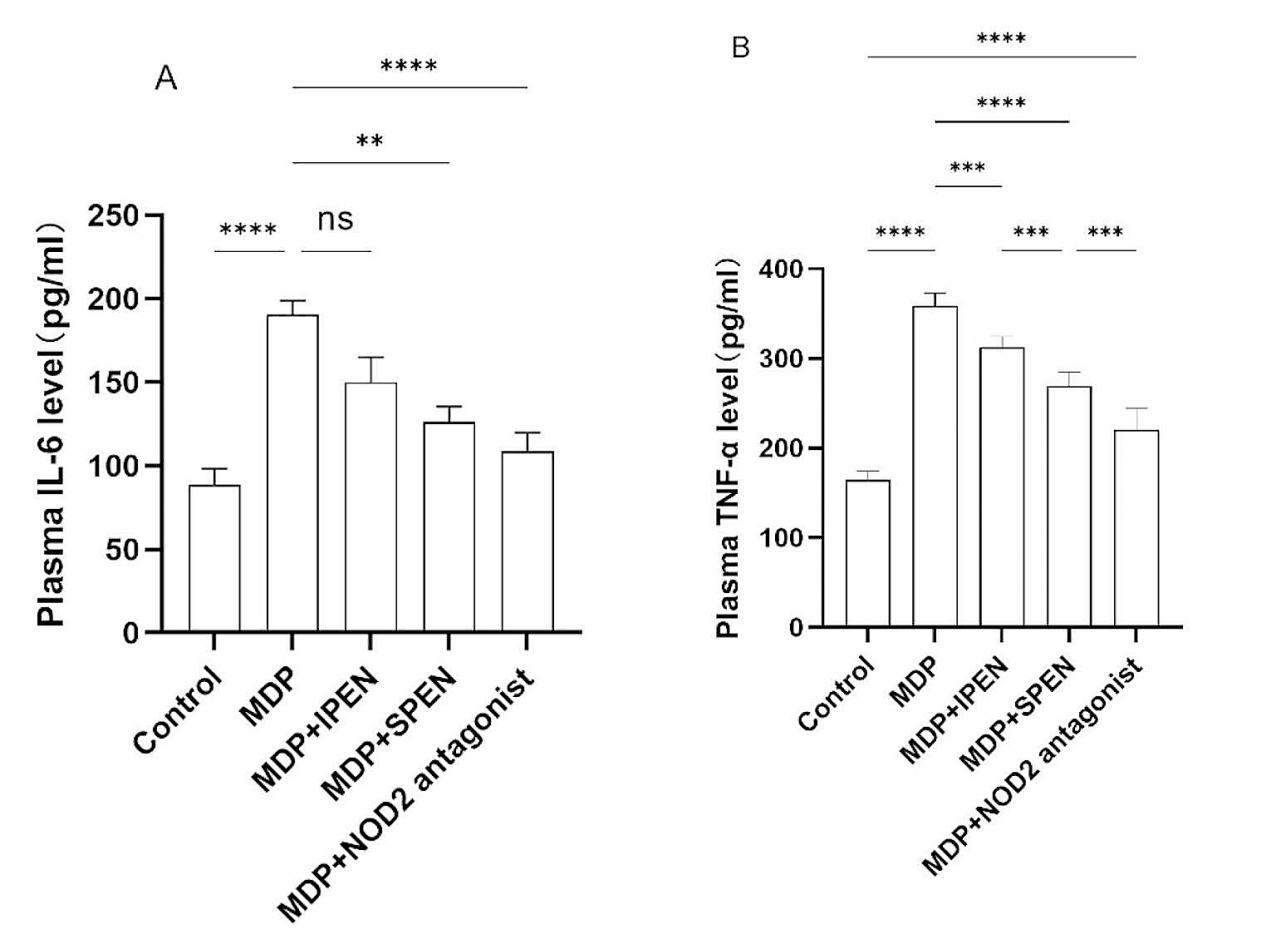
The expression of the proinflammatory factors IL-6 and TNF-α in the plasma of the different groups, was determined using ELISA. (**A-B**). Data were expressed as mean ± SD, *n* = 6; *P < 0.05; **P < 0.01; and ***P < 0.001; ****P < 0.0001

Rats experienced a systemic inflammatory response after being gavaged with MDP. To explore whether IL-6 and TNF-α were influenced by SPEN treatment, we determined their levels using an ELISA. Administration of MDP with SPEN group inhibited the release of proinflammatory factors in plasma more obviously than MDP with IPEN group, which was increased in rats gavaged with MDP. Administration of MDP with NOD2 antagonist group had the same level of IL-6 expression as MDP with SPEN group but lower in TNF-α expression than MDP with SPEN group (Fig. 2A-B).

### SPEN attenuates the MDP-induced pulmonary inflammatory response

**Fig. 3:**
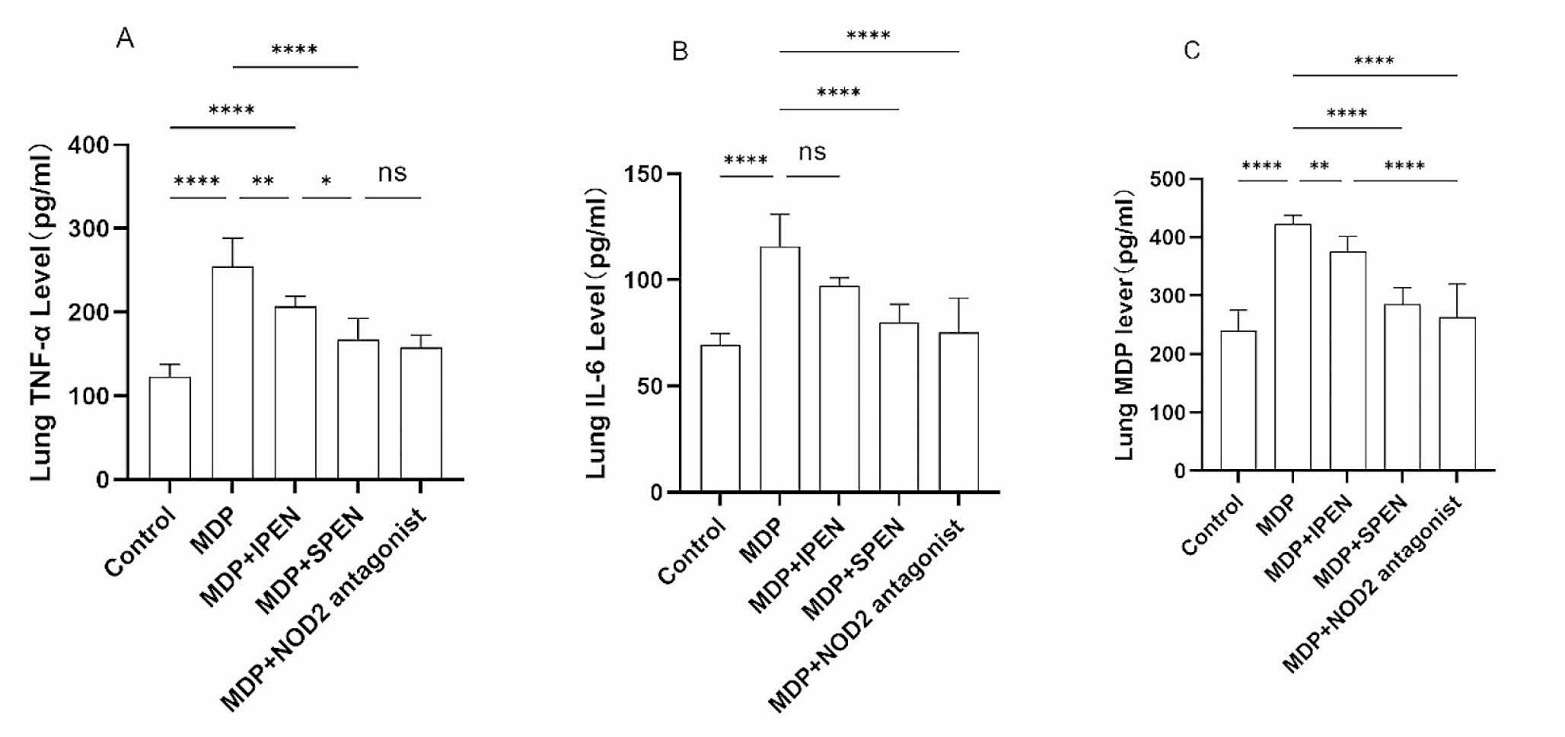
The expression of lung tissue proinflammatory factors IL-6, TNF-α, and MDP in each group was determined using ELISA. (**A-C**). Data were expressed as mean ± SD, *n* = 6; *P < 0.05; **P < 0.01; ***P < 0.001; ****P < 0.0001

To investigate whether the administered MDP migrates from the gut to the lung tissue and induces proinflammatory responses, we measured the expression of IL-6, TNF-α, and MDP in the lung tissue by means of ELISA. The expression of MDP increased in pulmonary after gavage of MDP. IL-6, TNF-α, and MDP expression in lung tissue were decreased in both the MDP with SPEN group and the MDP with IPEN group compared to the MDP group, but the decrease was more pronounced in the SPEN group. No significant difference in IL-6, TNF-α, or MDP expression was found between the MDP with SPEN group and the MDP with NOD2 antagonist group. (Fig. 3.)

### SPEN antagonizes MDP by downregulating PepT1

**Fig. 4:**
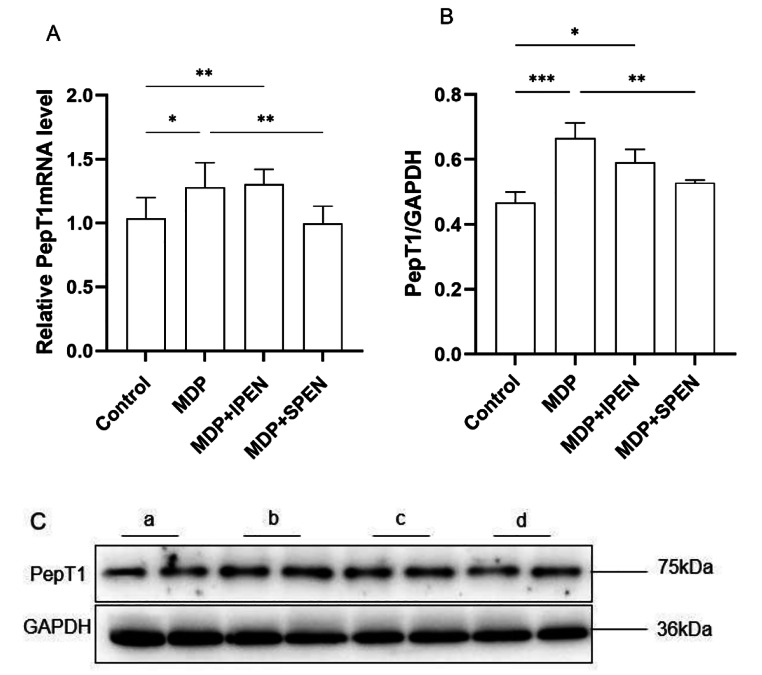
Determination of the level of expression of the PepT1 mRNA in the different groups (**A**). Western blotting was used to determine PepT1 protein levels. (**B-C**). Data were expressed as mean ± SD, *n* = 6; *P < 0.05; **P < 0.01; ***P < 0.001. a: control, b: MDP, c: MDP + IPEN, d: MDP + SPEN

Next, we determined whether SPEN antagonized MDP by competitively binding to PepT1. qRT-PCR and Western blot assays were used to analyze PepT1 expression in intestinal sections. PepT1 expression was upregulated after MDP treatment and downregulated in the MDP with SPEN group. These data indicated that SPEN competed with MDP for binding to PepT1, whereas IPEN did not (Fig. 4).

### SPEN reduces MDP-induced mitochondrial damage and autophagy

**Fig. 5:**
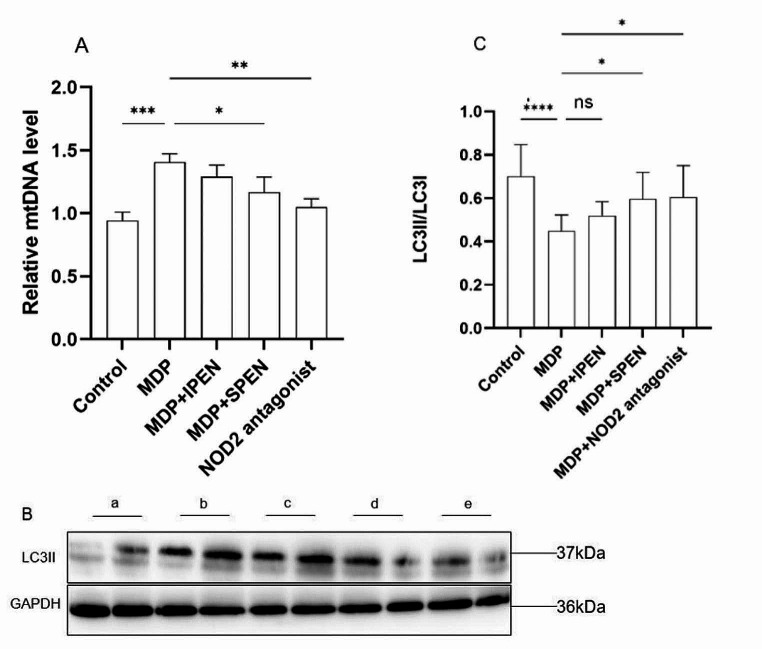
The expression of mtDNA was detected in different groups (**A**), and Western blotting was used to determine LC3II protein levels (**B-C**). Data were expressed as mean ± SD, *n* = 6; ns: no significance; *P < 0.05; **P < 0.01; ***P < 0.001; ****P < 0.0001. a: control, b: MDP, c: MDP + IPEN, d: MDP + SPEN, e: MDP + NOD2 antagonist

In order to explore the MDP’s effects on mitochondrial damage and autophagy in small intestinal cells, as well as the changes in mitochondrial damage and autophagy after SPEN intervention, we used PCR to test the mtDNA level and a western blot assay to detect the level of LC3II. We found that MDP aggravated mitochondrial damage in small intestinal mucosal epithelial cells, leading to a decrease in autophagy. The expression of mtDNA was markedly reduced in the MDP + SPEN group and the MDP + NOD2 antagonist group, whereas the decrease in mtDNA expression was not significant in the MDP + IPEN group. The expression of LC3II increased in the MDP + SPEN group and the MDP + NOD2 group, but not in the MDP + IPEN group. ((Fig. 5).

### SPEN reduces MDP-induced mitochondrial damage and autophagy in the jejunum cells through the NOD2/Beclin-1 pathway

**Fig. 6:**
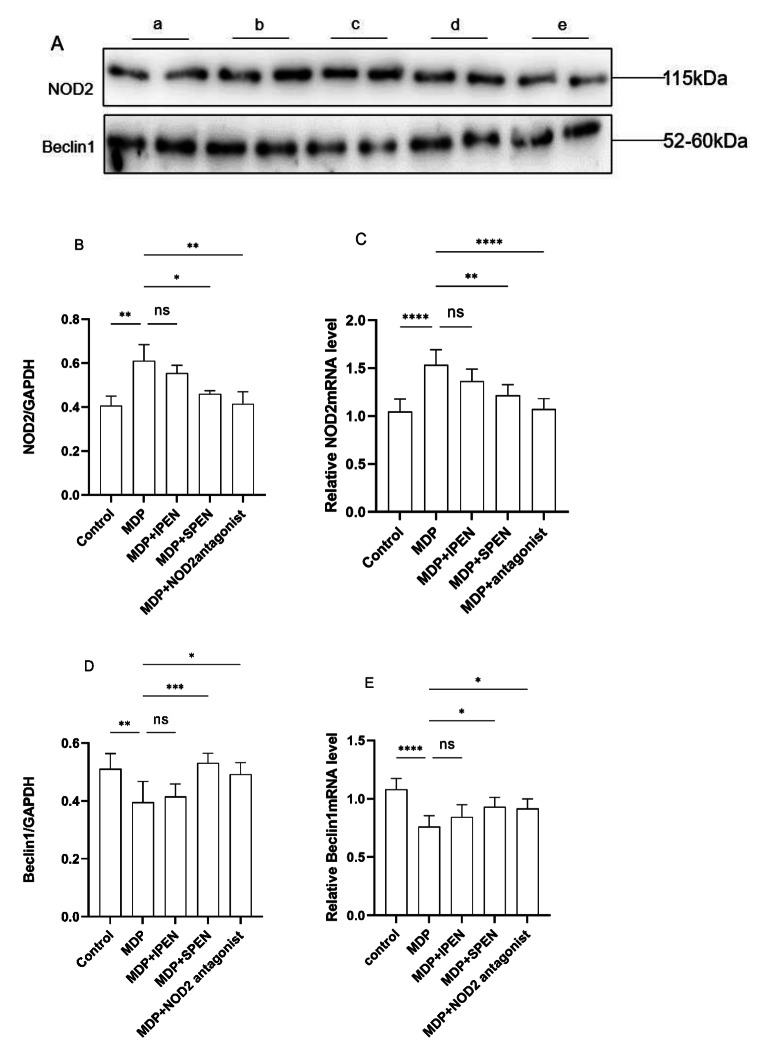
Western blotting was used to determine the NOD2 and Beclin-1protein levels (**A-B, D**). The expression of relative NOD2 mRNA and Beclin-1 mRNA were detected in different groups (**C, E**). Data were expressed as mean ± SD, *n* = 6; ns: no significance; *P < 0.05; **P < 0.01; ***P < 0.001; ****P < 0.0001. a: control, b: MDP, c: MDP + IPEN, d: MDP + SPEN, e: MDP + NOD2 antagonist

In order to explore whether SPEN reduces MDP-induced mitochondrial damage and autophagy in the jejunum cells via the NOD2/beclin-1 pathway, we measured NOD2, beclin-1 levels. MDP increased NOD2 levels, whereas SPEN and the NOD2 antagonist reduced the increase in their levels caused by MDP. Additionally, MDP decreased beclin-1 and LC3-II levels, and SPEN and the NOD2 antagonist reversed this effect. (Fig. 6).

### SPEN reduces MDP-induced apoptosis in small intestinal epithelial cells through the NOD2/beclin-1 pathway

**Fig. 7:**
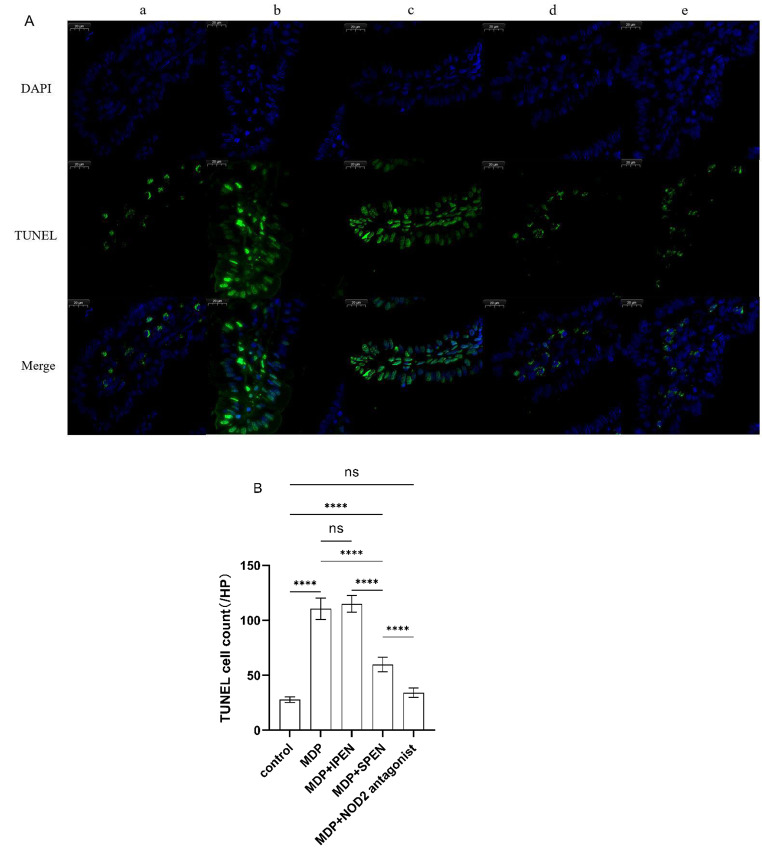
TUNEL images (500x) in the small intestine of mice subjected to MDP injury (**A**). The TUNEL cell count of each group (**B**). a: control, b: MDP, c: MDP + IPEN, d: MDP + SPEN, e: MDP + NOD2 antagonist. ****P < 0.0001

To further examine the relationship between MDP and apoptosis, we conducted TUNEL staining. Intestinal epithelial cells treated with MDP had a significant increase in apoptosis that was decreased by SPEN and the NOD2 antagonist. However, IPEN did not decrease apoptosis. (Fig. 7)

## Discussion

NOD2 is a cytoplasmic receptor activated by intracellular MDP that mediates innate immune responses and triggers proinflammatory responses [22, 23]. MDP enters cells through four pathways. One of the most important transport pathways is mediated by PepT1, which is expressed on the apical plasma membrane of intestinal epithelial cells [24], and delivers extracellular MDP to the cells. PepT1 is mainly expressed in the small intestines. It is a transporter of short peptides for metabolic purposes, such as dipeptides and tripeptides, and various other peptides, such as β-lactam antibiotics, protease inhibitors, antiviral drugs, and specific angiotensin-converting enzyme inhibitors [25]. MDP-mediated damage to the intestine begins with the cross-cellular pathway mediated by PepT1. Gly-Gly competes with PepT1 to reduce the translocation of intestinal bacterial products, thereby improving the intestinal inflammatory response. This effect is mediated through the NOD2/RIP2 pathway [5]. These provide new ideas for the selection of enteral nutrition in sepsis.

In this study, we used SPEN, which consists mainly of hydrolyzed whey protein. Protein is hydrolyzed into 80% short peptides and 20% free amino acids. In accordance with the characteristics of the PepT1 transport peptide, SPEN competitively inhibits the function and expression of PepT1 after MDP perfusion. Our investigation also yielded this result. We found that PepT1 expression was significantly downregulated after administering SPEN compared with the MDP group, but was not significantly affected in the MDP with IPEN group. This suggests that PepT1 preferentially translocates short peptide nutrients in the presence of short peptide nutrients, and that PepT1 expression increases in the absence of enteral nutrients, thereby increasing the transport of MDPs into the cell, thereby activating the NOD2 receptor and causing a series of responses. The reason why Pept1 expression was also upregulated in the MDP with IPEN group may be due to the inability of IPEN to be converted into short peptides by digestive enzymes within a short period, or to the lack of intestinal trypsin due to MDP. Further investigation of the expression of short peptides in the intestinal fluid and the expression of digestive enzymes will help to solve this mystery. It has also been previously shown that PepT1 expression is increased in the intestine in the absence of nutrition. For instance, it has been shown that fasting reduces the length and density of small intestinal villi, decreases cell proliferation, increases apoptosis, and leads to atrophy of the intestinal mucosa [26]. However, in mammals, refeeding and adequate nutrient availability suppress PepT1 expression. Compared with samples from randomly fed rats, amino acid supplementation reduces PepT1 protein expression in the jejunal mucosa by 30% [27].

Our findings were that the expression of NOD2 was significantly downregulated in the MDP with SPEN group, which was the same effect observed in the MDP with NOD2 antagonist group, indicating that SPEN inhibited PepT1 expression and suppressed MDP translocation through competition with PepT1, thereby reducing NOD2 expression. Moreover, serum IL-6 and TNF-α in the MDP with SPEN group decreased compared with the MDP with IPEN group. HE staining showed that intestinal mucosal damage was also reduced in the MDP with SPEN group compared to the MDP group, whereas damage to the jejunum mucosa in the MDP with IPEN group was not significantly reduced. This suggests that the MDP we gavaged initiated the NOD2 signaling pathway after transferring into the cells. The signaling pathway of NOD2 has been widely characterized. After activation induced by MDP, NOD2 self-aggregates through the LRR domain and recruits downstream receptor molecule kinase receptor-interacting protein 2 (RIP2) through homophilic CARD–CARD interactions [28, 29]. Activating RIP2 leads to ubiquitination of NF-κB, activation of the IκB kinase (IKK) complex, and phosphorylation of NF-κB inhibitor-α, which inhibits NF-κB translocation to the nucleus and initiates transcription of proinflammatory genes, including cytokines, growth factors, and factors that stimulate immune cells [30, 31]. RIP2 acts on TGF (transforming growth factor) through IKK complex-β activated kinase 1 and activates MAP kinase and transcription factor activating protein 1, which engage in cellular growth, differentiation, and apoptosis [28, 32].

To understand how SPEN regulates mitochondrial damage, apoptosis, and autophagy through NOD2, we examined the expression of mtDNA, Becin-1, LC3-II, and intestinal mucosal apoptosis. In our study, in terms of mitochondrial damage, the MDP with SPEN group showed a reduction in mitochondrial damage compared to the MDP group, whereas the MDP with IPEN group showed no significant reduction in mitochondrial damage. And Beclin-1 and LC3-II levels in the MDP with SPEN group were clearly elevated versus the MDP group, indicating an increase in intestinal mucosal autophagy. Intestinal mucosal apoptosis in the MDP with SPEN group was also significantly reduced. Similarly, the MDP with NOD2 antagonist group also had increased levels of Becin-1 and LC3-II and reduced apoptosis. These data suggest that SPEN downregulates NOD2 through the PepT1-NOD2-beclin-1 pathway, reduces apoptosis, and alleviates mitochondrial damage and intestinal injury. Increased expression of Beclin1 promotes autophagy.

Autophagy is a type II programmed cell death. It is a typical dynamic life process in which cells use lysosomal degradation to selectively remove their damaged, senescent, or excess biomolecules and organelles, releasing free small molecules for cell recycling. Autophagy is considered a self-protective mechanism of the organism and plays a vital role in innate and adaptive immunity, adaptation to starvation, and the elimination of intracellular pathogens. The roles of NOD2 in autophagy have been recently revealed and are still the subject of debate. It was found that NOD2 passes through the independent adapter RIP2, and the mechanism of NF-κB involves the recruitment of the autophagy protein TG16L1 to the bacterial entry site plasma membrane [33]. Additionally, knockdown of TG16L1 results in a targeted upregulation of the NOD2 response, and thus establishes a fat part of TG16L1 as a negative regulator of the NOD2/RIP2 signaling pathway [34]. Nevertheless, a recent study of boswellic acid (BA) and apigenin (APG) in the treatment of MTX-induced neuronal and renal injury found that BA and APG exerted anti-inflammatory effects by activating autophagy and inhibiting NOD-2 and p-NF-κB p65 to reduce TNF-α, IL-6, and NLRP3/IL-1β [35]. This suggests that, like our experimental results, inhibition of NOD2 expression also increases autophagy. Our other studies have also shown that MDP translocation reduces mitochondrial autophagy by regulating the NOD2/AMPK/LC3 pathway, leading to mitochondrial dysfunction. SPEN prevents MDP-induced impairment of intestinal epithelial mitochondrial function during sepsis [36].

The two most fundamental processes in cells—autophagy and apoptosis—are suicide processes, that result in the elimination of redundant, injured, and unneeded cells and organelles. When these processes are disrupted, the results may be catastrophic and become the major contributor to death and disability in many diseases [37]. A crosstalk mechanism between autophagy and apoptosis is the cleavage of Beclin-1 by apoptotic caspases, leading to its functional inactivation and reduced autophagy. Caspase-3 and − 8 cleave beclin-1 at D124 and D149 or D133 and D146 sites under various stimuli, including TRAIL (tumor necrosis factor-related apoptosis-inducing ligand) [38]. These data indicate that autophagy stops during apoptosis, while beclin-1 is deployed to amplify cell death. Min JJ’s study showed that butylphthalide activated the Nrf2/HO-1 pathway, inhibited the NOD2/MAPK/NF-κB pathway, increased cell viability, and antagonized apoptosis [39]. Our study showed that rats fed with MDP had reduced autophagy and increased apoptosis, and SPEN reduced MDP-induced apoptosis. Consistent with this finding, SPEN balances cell synthesis and decomposition by increasing autophagy and reducing apoptosis, promoting physical recovery. The NOD2 antagonist group also had increased autophagy and reduced apoptosis. Therefore, SPEN balances autophagy and apoptosis through the NOD2-beclin-1 pathway.

In defense against pathogens and endotoxins, inflammation, and bacterial translocation, the intestinal barrier plays an important role. Intestinal mucosal macrophages and immune cells kill most translocated bacteria. However, unkillable microorganisms and their products may reach the lung and activate alveolar macrophages, with the potential for lung damage [40]. Dickson et al. [41] found that the lungs of the mouse model of sepsis and the bronchoalveolar lavage fluid from patients with acute respiratory distress syndrome contained live intestinal bacteria, indicating that local or systemic inflammation may mediate intestinal microflora displacement and disrupt lung microecological homeostasis. Therefore, intestinal microecological imbalance increases intestinal permeability, which may promote the direct transfer of bacteria or their products to the lungs, further aggravating the inflammatory response of the lungs. We also examined relevant indicators for the effects of short peptides on the lungs and first found that MDP was increased in the lungs of the MDP group, indicating that MDP translocated to the lungs through the blood transport pathway, leading to increased inflammation in lung tissue. SPEN reduced MDP translocation and alleviated inflammation in lung tissue, but the mechanism requires further research.

Short peptides are attracting attention for their broad range of applications, as they are distinct biological molecules that combine the advantages of classic small molecules and mature proteins. During the pandemic, some short peptide study contributions concentrated on SARS-CoV-2-related research. Based on the natural structure of angiotensin-converting enzyme 2, Odolczyk et al. [42] have designed short peptides that can be used as potential inhibitors of the protein-protein interaction of the SARS-CoV-2 spike proteins. These peptides have a high affinity for binding to viral proteins. In another study [43], by screening the Antimicrobial Peptide Database (APD), fifteen peptides were selected on basis of their physicochemical and antiviral properties, and then by docking analysis to identify the new potentially active AMPs with antiviral properties against SARS-CoV-2, but their therapeutic efficacy still needs to be experimentally validated. Other researchers have investigated the important role of short peptides in effectively fighting bacterial and fungal infections. The antimicrobial activity of cationic antimicrobial peptides extracted from cecropin D-like (ΔM2) against multi-drug resistant bacteria was evaluated by Rivera-Sanchez et al. This antimicrobial peptide showed strong bactericidal effects against *Klebsiella pneumoniae* and *Pseudomonas aeruginosa* [44]. The short peptides in the above studies were synthetic peptides, not food-derived short peptides. Research on SPEN is scarce. Zhang et al. found that SPEN improves secondary downregulation of intestinal mucosal microcirculation in acute pancreatitis, which may help alleviate mucosal inflammation, maintain mechanical barriers and mucosal immunity, correct systemic immune suppression, and play a role in preventing symbiotic bacterial translocation after acute pancreatitis [45]. These studies on short peptides clearly demonstrate the new trends in the field of short peptides.

Our study also has certain limitations. MDP and SPEN coadministration in this study only indicated that SPEN might have an anti-inflammatory effect, promote autophagy, and prevent apoptosis in the early stages of intestinal infections or bacterial end-product translocation. However, further clinical research is needed to determine which stage of clinical disease development these apply to. Also, the difference between SPEN pretreatment and coadministration needs further study. In addition, we did not test the changes of MDP, short peptides, and trypsin in the intestinal fluid before and after the experiment to clarify the digestive capacity of the intestine. We did not test intestinal barrier function to further define the pathway of MDP translocation.

## Conclusion

SPEN improves intestinal and systemic inflammatory responses and mitochondrial damage caused by MDP, possibly by promoting autophagy and reducing apoptosis through the PepT1-NOD2-beclin-1 pathway. The intestinal bacterial product MDP translocates to the lungs, leading to an increase in the pneumonia response. SPEN improves the pneumonia response caused by MDP.

## Data Availability

The data analyzed for this study are available from the first author or corresponding author.

## References

[1] Edwin AD (2002) Bacterial translocation or lymphatic drainage of toxic products from the gut: what is important in human beings? Surgery 131:241–244. 10.1067/msy.2002.116408 11894026 10.1067/msy.2002.116408

[2] Gatt M, Reddy BS, MacFie J (2007) Review article: bacterial translocation in the critically ill–evidence and methods of prevention. Aliment Pharmacol Ther 25:741–757. 10.1111/j.1365-2036.200603174. x 17373913 10.1111/j.1365-2036.2006.03174.x

[3] Merlin D, Steel A, Gewirtz AT et al (2008) hPepT1-mediated epithelial transport of bacteria-derived chemotactic peptides enhances neutrophil-epithelial interactions. J Clin Invest 102:2011–2018. 10.1172/JCI4179 10.1172/JCI4179 PMC509154 9835627

[4] Chikako O, Yuen-Joyce L, and Koichi S. K (2011) Muramyl Dipeptide and its derivatives: peptide adjuvant in Immunological disorders and Cancer Therapy. Curr Bioact Compd 7(3):180–197. 10.2174/157340711796817913 22180736 10.2174/157340711796817913 PMC3241611

[5] Adam A, Ciorbaru R, Ellouz F, Petit JF, Lederer E (1974) Adjuvant activity of monomeric bacterial cell wall peptidoglycans. Biochem Biophys Res Commun 56(3):561–567. 10.1016/0006-291x(74)90640-8 4597063 10.1016/0006-291x(74)90640-8

[6] White PJ, Gilvarg C (1977) A teichuronic acid containing rhamnose from cell walls of Bacillus megaterium. Biochemistry. 31;16(11):2428-35. 10.1021/bi00630a018 10.1021/bi00630a018 405039

[7] Ma GG, Shi B, Liu JQ et al (2015) Nod2–Rip2 signaling contributes to Intestinal Injury Induced by Muramyl Dipeptide Via Oligopeptide Transporter in rats. Dig Dis Sci 60:3264–3270. 10.1007/s10620-015-3762-1 26138652 10.1007/s10620-015-3762-1

[8] Strober W, Murray PJ, Kitani A et al (2006) Signaling pathways and molecular interactions of NOD1 and NOD2. Nat Rev Immunol 6:9–20. 10.1038/nri1747 16493424 10.1038/nri1747

[9] Shaw MH, Kamada N, Warner N et al (2011) The ever-expanding function of NOD2: autophagy, viral recognition, and T cell activation. Trends Immunol 32(2):73–79. 10.1016/j.it.2010.12.007 21251876 10.1016/j.it.2010.12.007 PMC3056277

[10] Jiang W, Wang XQ, Zeng BH et al (2013) Recognition of gut microbiota by NOD2 is essential for the homeostasis of intestinal intraepithelial lymphocytes (IEL). J Exp Med 210(11):2465–2476. 10.1084/jem.20122490 24062413 10.1084/jem.20122490 PMC3804938

[11] Negroni A, Pierdomenico M, Cucchiara S et al (2018) Salvatore Cucchiara NOD2 and inflammation: current insights. J Inflamm Res 11:49–60. 10.2147/JIR.S137606 29483781 10.2147/JIR.S137606 PMC5813767

[12] Negroni A, Colantoni E, Vitali R et al (2016) NOD2 induces autophagy to control AIEC bacteria infectiveness in intestinal epithelial cells. Inflamm Res 65(10):803–813. 10.1007/s00011-016-0964-8 27335178 10.1007/s00011-016-0964-8

[13] Panzer AR, Lynch SV (2015) Influence and effect of the human microbiome in allergy and asthma. Curr Opin Rheumatol 27(4):373–380. 10.1097/BOR.0000000000000191 26002029 10.1097/BOR.0000000000000191

[14] Girardin SE, Boneca IG, Viala J et al (2003) Nod2 is a general sensor of peptidoglycan through muramyl dipeptide (MDP) detection. J Biol Chem 278:8869–8872. 10.1074/jbc.C200651200 12527755 10.1074/jbc.C200651200

[15] Keestra-Gounder AM, Byndloss MX, Seyffert N et al (2016) NOD1and NOD2 signaling links ER stress with inflammation. Nature 532(7599):394–397. 10.1038/nature17631 27007849 10.1038/nature17631 PMC4869892

[16] Copetti T, Bertoli C, Dalla E et al (2009) p65/RelA modulates BECN1 transcription and autophagy. Mol Cell Biol 29(10):2594–2608. 10.1128/MCB.01396-08 19289499 10.1128/MCB.01396-08 PMC2682036

[17] Pattingre S, Tassa A, Qu X et al (2005) Bcl-2 antiapoptotic proteins inhibit Beclin1-dependent autophagy. Cell 122(6):927–939. 10.1016/j.cell.2005.07.002 16179260 10.1016/j.cell.2005.07.002

[18] Pimkina J, Humbey O, Zilfou JT et al (2009) ARF induces autophagy by virtue of interaction with Bcl-x. J Biol Chem 284(5):2803–2810. 10.1074/jbc.M804705200 19049976 10.1074/jbc.M804705200 PMC2631963

[19] Thorburn AN, McKenzie CI, Shen S et al (2015) Evidence that Asthma is a developmental origin disease influenced by maternal diet and bacterial metabolites. Nat Commun 6:7320–7333. 10.1038/ncomms8320 26102221 10.1038/ncomms8320

[20] Felipe MG, Javiera SA, Bárbara MS et al (2022) Distal consequences of mucosal infections in intestinal and lung inflammation. Front Immunol 13(3):877533. 10.3389/fimmu.2022.877533 35572549 10.3389/fimmu.2022.877533 PMC9095905

[21] Chiu CJ, McArdle AH, Brown R, Scott HJ, Gurd FN (1970) Intestinal mucosal lesion in low-flow states. I. A morphological, hemodynamic, and metabolic reappraisal. Arch Surg 101(4):478–483. 10.1001/archsurg.1970.01340280030009 5457245 10.1001/archsurg.1970.01340280030009

[22] Takeuchi O, Akira S (2010) Pattern recognition receptors and inflammation. Cell 140(6):805–820. 10.1016/j.cell.2010.01.022 20303872 10.1016/j.cell.2010.01.022

[23] Kawai T, Akira S (2009) The roles of TLRs, RLRs and NLRs in pathogen recognition. Int Immunol 21(4):317–337. 10.1093/intimm/dxp017 19246554 10.1093/intimm/dxp017 PMC2721684

[24] Wang YQ, Hu YJ, Li P et al (2018) Expression and regulation of proton-coupled oligopeptide transporters in colonic tissue and immune cells of mice. Biochem Pharmacol 148:163–173. 10.1016/j.bcp.2017.12.025 29305856 10.1016/j.bcp.2017.12.025 PMC5801143

[25] Rubio-Aliaga I, Daniel H (2008) Peptide transporters and their roles in physiological processes and drug disposition. Xenobiotica 38(7–8):1022–1042. 10.1080/00498250701875254 18668438 10.1080/00498250701875254

[26] Chappell VL, Thompson MD, Jeschke MG et al (2003) Effects of incremental starvation on gut mucosa. Dig Dis Sci 48:765–769. 10.1023/a:1022849112100 12741469 10.1023/a:1022849112100

[27] Ogihara H, Suzuki T, Nagamachi Y, Inui K, Takata K (1999) Peptide transporter in the rat small intestine: ultrastructural localization and the effect of starvation and administration of amino acids. Histochem J 31:169–174. 10.1023/a:1003515413550 10421416 10.1023/a:1003515413550

[28] Girardin SE, Boneca IG, Viala J et al (2003) Nod2 is a general sensor of peptidoglycan through muramyl dipeptide (MDP) detection. J Biol Chem 278(11):8869–8872. 10.1074/jbc.C200651200 12527755 10.1074/jbc.C200651200

[29] Park JH, Kim YG, McDonald C et al (2007) RICK/RIP2 mediates innate immune responses induced through Nod1 and Nod2 but not TLRs. J Immunol 178(4):2380–2386. 10.4049/jimmunol.178.4.2380 17277144 10.4049/jimmunol.178.4.2380

[30] Rahighi S, Ikeda F, Kawasaki M et al (2009) Specific recognition of linear ubiquitin chains by NEMO is important for NF-κB activation. Cell 136(6):1098–1109. 10.1016/j.cell.2009.03.007 19303852 10.1016/j.cell.2009.03.007

[31] Jiang X, Chen ZJ (2011) The role of ubiquitylation in immune defense and pathogen evasion. Nat Rev Immunol 12(1):35–48. 10.1038/nri3111 22158412 10.1038/nri3111 PMC3864900

[32] Karin M (1995) The regulation of AP-1 activity by Mitogen-activated protein kinases. J Biol Chem 351(1336):127–134. 10.1074/jbc.270.28.16483 10.1074/jbc.270.28.16483 7622446

[33] Travassos LH, Carneiro LA, Ramjeet M et al (2010) Nod1 and Nod2 direct autophagy by recruiting ATG16L1 to the plasma membrane at the site of bacterial entry. Nat Immunol 11(1):55–62. 10.1038/ni.1823 19898471 10.1038/ni.1823

[34] Sorbara MT, Ellison LK, Ramjeet M et al (2013) The protein ATG16L1 suppresses inflammatory cytokines induced by the intracellular sensors Nod1 and Nod2 in an autophagy-independent manner. Immunity 39(5):858–873. 10.1016/j.immuni.2013.10.013 24238340 10.1016/j.immuni.2013.10.013

[35] Sarah AA, El-Aal SM, El-Sayyad AA, El-Gazar et al (2024) Boswellic acid and apigenin alleviate methotrexate-provoked renal and hippocampal alterations in rats: targeting autophagy, NOD-2/NF-κB/NLRP3, and connexin-43. Int Immunopharmacol 15:134112147. 10.1016/j.intimp.2024.112147 10.1016/j.intimp.2024.112147 38718656

[36] Zhao LJ, Dai XY, Ye YW, Pang XF, Shi B et al (2024) Muramyl dipeptide causes mitochondrial dysfunction and intestinal inflammatory cytokine responses in rats. Shock. 2024 Mar 28. 10.1097/SHK.0000000000002369 10.1097/SHK.0000000000002369 38546380

[37] Prerna K, Dubey VK (2022) Repurposing of FDA-approved drugs as autophagy inhibitors in tumor cells. J Biomol Struct Dyn 40(13):5815–5826. 10.1080/07391102.2021.1873862 33467992 10.1080/07391102.2021.1873862

[38] Tran S, Fairlie WD, Lee EF (2021) BECLIN1: protein structure, function and regulation. Cells 10:1522. 10.3390/cells10061522 34204202 10.3390/cells10061522 PMC8235419

[39] Min JJ, Chen Q, Pan MX, Liu T, Gu Q, Zhang DW, Sun R (2023) Butylphthalide improves brain damage induced by renal ischemia-reperfusion injury rats through Nrf2/HO-1 and NOD2/MAPK/NF-κB pathways. Ren Fail 45(2):2259234. 10.1080/0886022X.2023.2259234 37732403 10.1080/0886022X.2023.2259234 PMC10515692

[40] Samuelson DR, Welsh DA, Shellito JE (2015) Regulation of lung immunity and host defense by the intestinal microbiota. Front Microbiol 6:1085. 10.3389/fmicb.2015.01085 26500629 10.3389/fmicb.2015.01085 PMC4595839

[41] Dickson RP, Singer BH, Newstead MW et al (2016) Enrichment of the lung microbiome with gut bacteria in sepsis and the acute respiratory distress syndrome. Nat Microbiol 1(10):16113. 10.1038/nmicrobiol.2016.113 27670109 10.1038/nmicrobiol.2016.113 PMC5076472

[42] Odolczyk N, Marzec E, Winiewska-Szajewska M, Pozna´nski J, Zielenkiewicz P (2021) Native structure-based peptides as potential protein–protein Interaction inhibitors of SARS-CoV-2 spike protein and human ACE2 receptor. Front Mol Biosci 28:9983014. 10.3389/fmolb.2022.983014 10.3390/molecules26082157 PMC8070189 33918595

[43] Liscano Y, Onate-Garzón J, Ocampo-Ibanez ID (2020) In silico discovery of antimicrobial peptides as an alternative to controlmSARS-CoV-2. Molecules 25:5535. 10.3390/molecules25235535 33255849 10.3390/molecules25235535 PMC7728342

[44] Rivera-Sanchez SP, Agudelo-Góngora HA, Onate-Garzon J et al (2020) Antibacterial activity of a cationic antimicrobial peptide against multidrug-resistant gram-negative clinical isolates and their potential molecular targets. Molecules 25(21):5035. 10.3390/molecules25215035 33142969 10.3390/molecules25215035 PMC7663601

[45] Huang L, Li G, Zhou B et al (2020) Clinical effects of total protein and short peptide enteral nutrition during recovery after radical gastrectomy. Asia Pac J Clin Nutr 29(2):239–244. 10.6133/apjcn.202007_29(2).0005 32674230 10.6133/apjcn.202007_29(2).0005

